# Case Report: Management of Recurrent Ovarian Squamous Cell Carcinoma With PD-1 Inhibitor

**DOI:** 10.3389/fonc.2022.789228

**Published:** 2022-03-09

**Authors:** Xiao-chen Song, Yong-xue Wang, Mei Yu, Dong-yan Cao, Jia-xin Yang

**Affiliations:** Department of Obstetrics and Gynecology, Peking Union Medical College Hospital, Chinese Academy of Medical Sciences & Peking Union Medical College, National Clinical Research Center for Obstetrics & Gynecologic Diseases, Beijing, China

**Keywords:** recurrence, PD-1 inhibitor, chemotherapy, immunotherapy combined therapy, ovarian squamous cell carcinoma

## Abstract

Malignant transformations, such as ovarian squamous cell carcinoma (SCC) in ovarian mature cystic teratoma (OMCT), are rare tumors. The management of recurrent disease is still a challenge, and the gene mutations involved remain unclear. We herein report a recurrent case of ovarian SCC with a PIK3CA gene variation and immunohistochemical staining of programmed death-ligand 1 (PD-L1) >10%. This patient achieved clinical remission after platinum-based effective chemotherapy and programmed death 1 (PD-1) immunotherapy.

## Introduction

Malignant transformation, such as ovarian squamous cell carcinoma (SCC) in ovarian mature cystic teratoma (OMCT), is a rare tumor. It exhibits a poor prognosis when diagnosed at advanced stages (1).

The gene mutations involved remain unclear, and the management of recurrent disease is still a challenge.

In recent years, immune checkpoint inhibitors (ICIs) have demonstrated great potential in treating a variety of cancers. To our knowledge, there is only one report in the literature of metastatic SCC arising from OMCT treated with an ICI (2). We herein report a recurrent case of ovarian SCC with a PIK3CA gene variation and immunohistochemical staining of programmed death-ligand 1 (PD-L1) >10%. This patient achieved clinical remission after platinum-based effective chemotherapy and programmed death 1 (PD-1) immunotherapy.

## Case Presentation

A 63-year-old woman (gravida 3, para 1) was referred to our hospital because of lower abdominal discomfort, and ultrasonography revealed an 8.7 * 8.0 cm heterogeneous adnexal mass in June 2018. The tumor markers were cancer antigen 125 (CA125) of 552.5 U/ml (<35) and SCC-associated antigen (Scc-Ag) of 9.4 ng/ml (<1.5). The human papillomavirus (HPV) screen of the cervix was negative before surgery. Due to the malignancy detected in frozen sections during the operation, laparoscopic hysterectomy plus bilateral salpingo-oophorectomy plus omentectomy plus pelvic lymph node dissection was performed. Postoperative pathologic examination indicated left OMCT with malignant transformation into moderately differentiated SCC. Metastases were not found in any other excised specimen. The stage was IC1 (cyst ruptured when taken out *via* vagina during operation) according to the International Federation of Gynecology and Obstetrics (FIGO) classification (3). Tumor markers decreased to normal after surgery. Three cycles of platinum-based chemotherapy (Taxol 175 mg/m^2^ D1 and cisplatin 70 mg/m^2^ D1, intravenously every 3 weeks) were given after surgery. Then, the patient received regular follow-up. The patient signed an informed consent that her data would be used for scientific purposes. The hospital’s ethics committee approved this study.

### First Recurrence

At 8 months after the last cycle of chemotherapy, serum Scc-Ag was elevated to 4.2 ng/ml, and PET-CT showed elevated uptake of ^18^F-fluorodeoxyglucose in pelvic parailiac vessels and the parasigmoid colon. Then, she returned in April 2019. Considering the multiple lesions in the pelvis, external beam radiation therapy (EBRT) (DT: 50 Gy/25 F) was first given to reduce the tumor burden. Subsequently, the patient was given three cycles of cisplatin plus 5-fluorouracil adjuvant chemotherapy (cisplatin 70 mg/m^2^ D1, 5-fluorouracil 1,000 mg/m^2^ D1–D4, intravenously every 3 weeks). Serum Scc-Ag decreased to normal after the first cycle of chemotherapy. The reduction of the tumor was satisfactory (remaining tumor <1 cm) after three cycles of chemotherapy.

**Figure 1:**
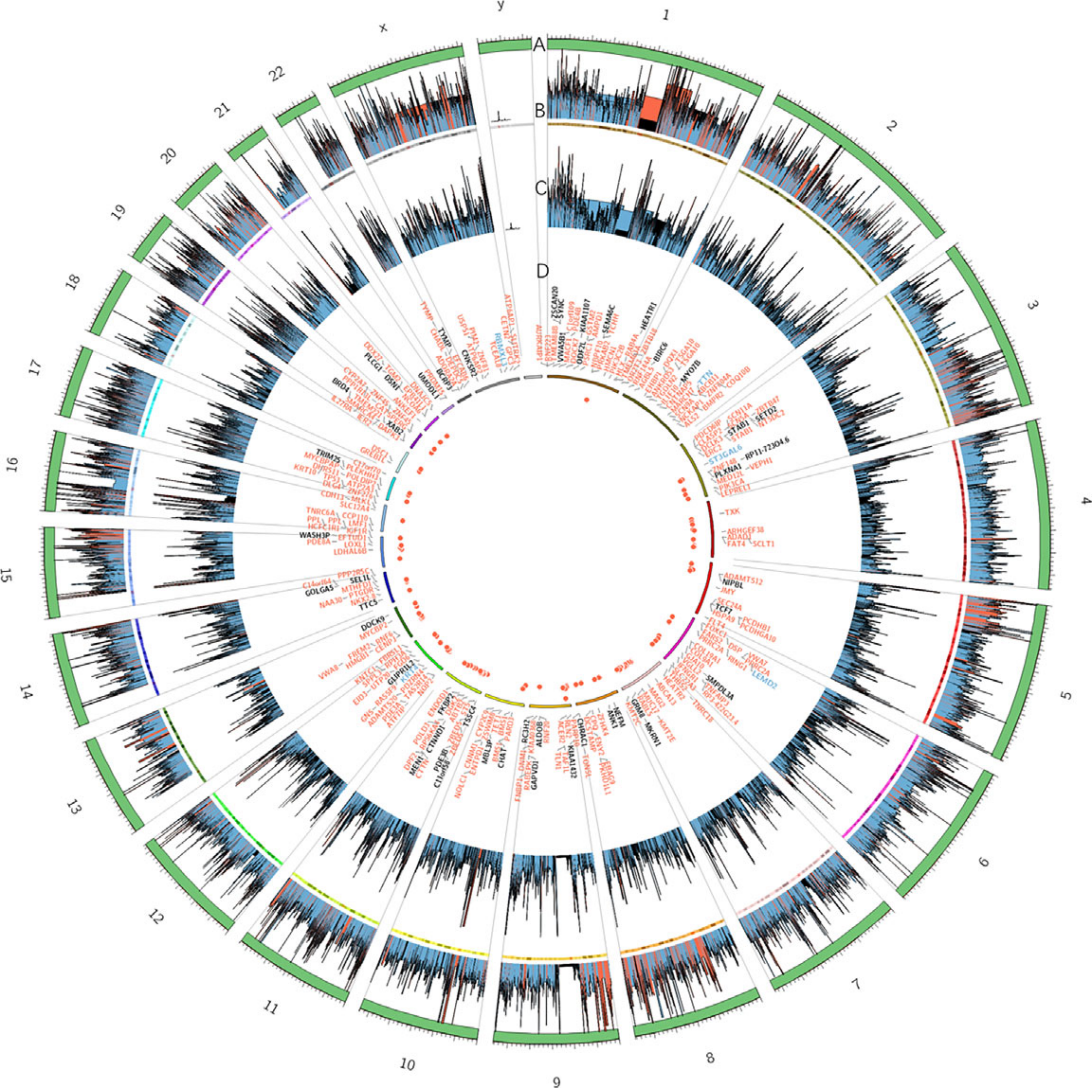
Summary of exome sequencing results of the tumor tissue in this patient displayed as a diagram. Each circle from the outer to inner is as follows: **(A)** trapping area. **(B)** Sequencing depth of tumor samples; different colors represent various depths: red ≥ 500, blue ≥ 100, and others are black. **(C)** Sequencing depth of control group samples. **(D)** Frequency of somatic mutations and genes related to exonic and splicing mutations: deletion/insertion mutations are blue, missense/nonsense synonymous mutations are red, and all others are black.

**Figure 2:**
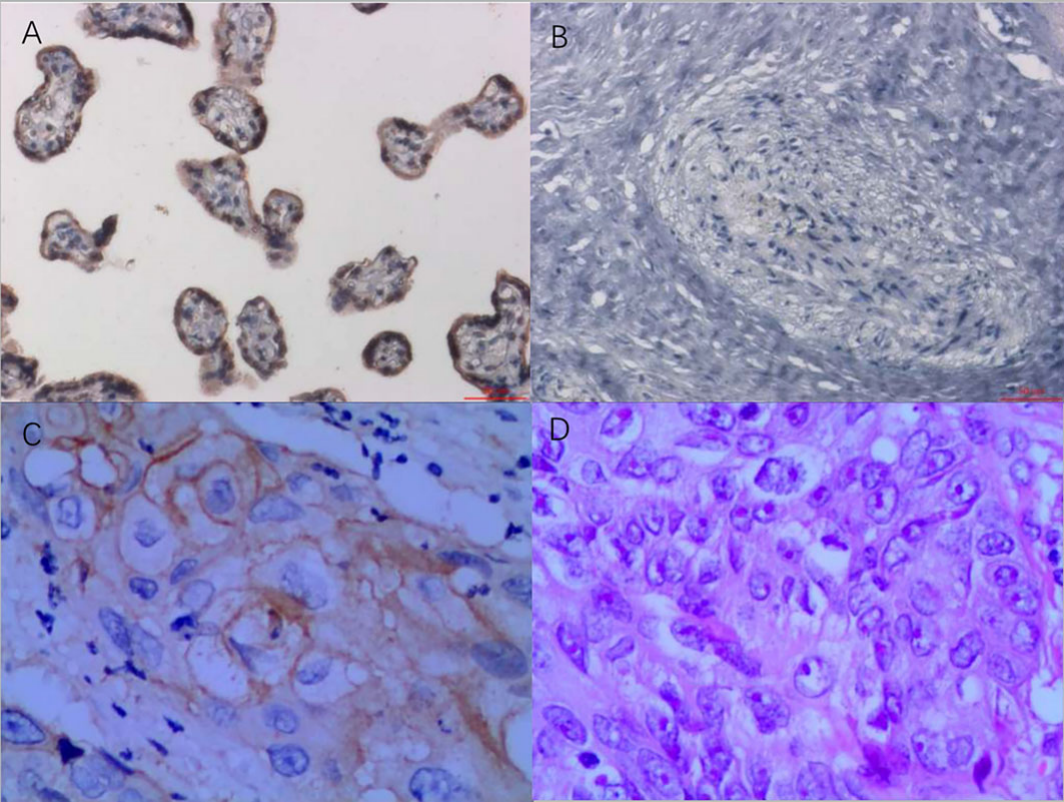
PD-L1 immunohistochemistry staining as a ratio of tumor cell membrane, VENTANA PD-L1 (SP263) Assay, Roche. **(A)** Positive control, 200×. **(B)** Negative control, 200×. **(C)** Immunohistochemistry of tumor tissue, 400×. **(D)** H&E staining of tumor tissue, 400×. Testing standard: only the staining ratio of the tumor cell membrane was calculated. The proportion of immunocyte staining was not calculated. The staining ratio of PD-L1 was divided into five levels, as follows: <1%, ≥1%, ≥5%, ≥10%, and ≥50%. PD-L1, programmed death-ligand 1.

Whole-exome sequencing of the patient identified 372 somatic mutation events (**Figure 1**). A total of 247 (247/372, 66.4%) were nonsynonymous single nucleotide variations, and 5 were disruptive insertion–deletion events. The immunohistochemical staining of tumor tissue was PD-L1-positive (>10%, **Figure 2**), and the tumor mutational burden was 7.280 muts/Mb. There were mutations in 14 known driver genes, including *PIK3CA*, *IFNGR1*, *MAPT*, *RPS6KA4*, *BMPR2*, *THBS2*, *PPP2R5C*, *CDKN1A*, *ENDOD1*, *BMPR2*, *FLT2*, *IGF1R*, *TLN1*, and *PARD3*. The PIK3CA gene mutation *p.H1047L* indicated that the patient may benefit from mTOR inhibitors.

After sufficient explanation and obtaining informed consent, the sirolimus oral pill was administered (2 mg per day). The adverse effect was slightly increased plasma transaminase, which recovered to normal after medication treatment. No other side effects were observed. However, at the seventh month of sirolimus, serum Scc-Ag was elevated again.

### Second Recurrence

**Figure 3:**
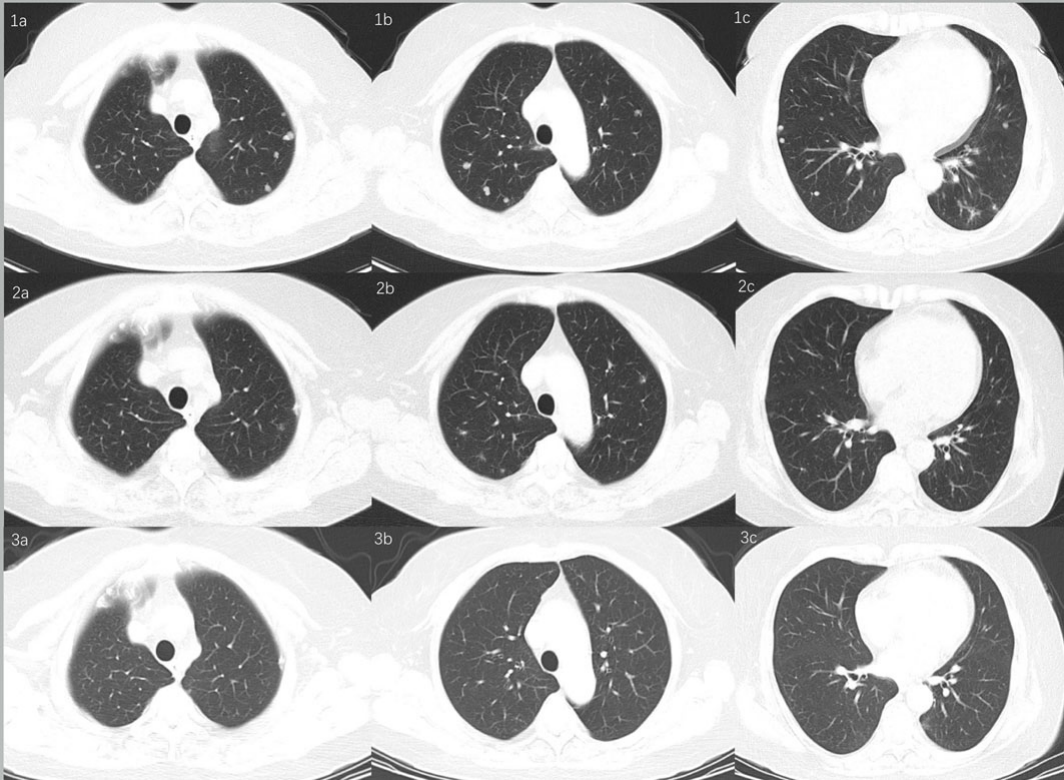
Chest CT scan showed lung metastasis. **(1a–1c)** *De novo* lung metastasis nodules. **(2a–2c)** Lung metastasis nodules reduced in size. **(3a–3c)** Nodules disappeared.

At the seventh month of sirolimus, serum Scc-Ag elevated to 3.7 ng/ml, and CT showed lung metastasis in April 2020 (**Figures 3, 1a–1c**). Considering the second recurrence of the tumor and given the tumor’s PD-L1 positivity, six cycles of platinum-based chemotherapy (Taxol 175 mg/m^2^ and cisplatin 70 mg/m^2^, intravenously every 3 weeks) plus PD-1 inhibitor (sintilimab injection, 200 mg, intravenously every 3 weeks) were administered after a discussion at the multidisciplinary tumor board. Sintilimab (Tyvyt^®^, Innovent Biologics Inc., Jiangsu, China) is a humanized monoclonal antibody that blocks the binding of PD-1 receptor to PD-L1 and PD-L2 and has shown efficacy against a wide variety of solid tumors. After six cycles of chemotherapy plus PD-1 inhibitor, the PD-1 inhibitor was administered every 3 weeks. Serum Scc-Ag decreased to normal after the first cycle of chemotherapy. A chest CT scan every 3 months showed that the lung metastasis nodules were narrowing (**Figures 3** and **2a–2c**), and in June 2021, there were no abnormal lesions (**Figures 3, 3a–3c**). The pelvic lesions were simultaneously reduced in size. To date, the patient continues on sintilimab at 13 months with stable disease. There has been no evidence of tumor recurrence.

## Discussion and Conclusions

Malignant transformation occurs in 1%–2% of OMCTs, and the majority of these (80%) are SCCs (1). For those patients, prompt diagnosis and individualized treatment are crucial for a better prognosis. Sakuma (4) reported that optimal cytoreductive surgery can bring a mean survival of 14 months, and in suboptimal cases with advanced-stage disease, it is 7.8 months. The current recommendation of postoperative platinum-based chemotherapy in ovarian SCC is related to its activity in ovarian cancer and gynecological SCCs (5). However, to date, the appropriate treatment for patients with recurrent ovarian SCC is still a difficult problem.

This patient had a 7-month progression-free survival (PFS) with sirolimus used as a maintenance treatment after chemotherapy at the first recurrence, and at the second recurrence, she had a PFS of more than 17 months with PD-1 inhibitor therapy. To date, the survival time has been more than 41 months. For this case with a second recurrence, there are several key issues that should be thoroughly discussed. First, the operation may have played a pivotal role as the initial treatment, but its role in the second cytoreductive surgery is uncertain. Second, the cytogenetic abnormalities might precede histological changes, and the increased copy number of PIK3CA and immunohistochemical positive staining for PD-L1 may be correlated with poor PFS. Thus, these abnormalities could be potential therapeutic targets for ovarian SCC, especially ICIs.

There is a rationale for ICI treatment in the management of ovarian cancer. However, the efficacy remains limited with a response rate of 10%–15% (6, 7). This may be due to a low tumor mutational burden and low PD-L1 expression (7). PD-L1 expression has emerged as one of the biomarkers that could predict sensitivity to ICIs (8). There are no data regarding the prevalence of PD-L1 expression in ovarian SCC, but studies of SCC of other primary sites have shown PD-L1 positivity ranging from 33% to 59% in squamous non-small cell lung cancer to 83.7% in cervical cancer (2, 9). In this patient, positive tumor PD-L1 expression (>10%) may be correlated with the responsiveness of ICI therapy.

Several studies have shown that the malignant transformation of OMCTs may be associated with genetic mutations. OMCT-associated SCC has a much higher overall mutational burden and more copy number alterations than OMCT (10). The most frequently altered genes in ovarian SCC were TP53 (80%), PIK3CA (52%), and CDKN2A (44%) (11). PIK3CA is a key oncogene located on chromosome 3, and *PIK3CA p.H1047L* is a hotspot mutation that lies within the PI3K/PI4K domain of the PIK3CA protein. *H1047L* confers a gain-of-function effect on the PIK3CA protein, as indicated by the increased phosphorylation of Akt and Mek 1/2, growth factor-independent cell survival, and transformation in cell cultures (12, 13). Mutations of PIK3CA will cause the PI3K enzyme to be continuously activated (14). It has been reported that a key signaling target of PD-1-mediated inhibition is the PI3K–Akt pathway (15, 16), and PD-1 can block the activation of PI3K by recruiting SHP-2 (16).

The immune system plays a significant role in ovarian cancer outcomes. Overexpression of the immune checkpoint receptor PD-L1 can lead to inhibition of an effective antitumor immune response (17). It has been reported that humanized monoclonal antibodies targeting PD-1 confer durable antitumor activity with acceptable safety and toxicity in patients with advanced PD-L1-positive ovarian cancer (18). Wu (2) reported the first case of a patient with metastatic SCC arising from OMCT who was successfully treated with pembrolizumab after progressing through platinum-based combination chemotherapy. Here, we present the clinical experience of a woman with recurrent SCC who responded to treatment with chemotherapy and the PD-1 inhibitor.

Because of tumor heterogeneity, gene mutations, which are the basis of targeted therapy in carcinomas with the same histological type, vary from case to case. Currently, personalized cancer medicine has become increasingly important for the treatment of malignancies. In addition to surgery and chemotherapy, targeted therapy could also benefit refractory cases.

In conclusion, this study is the first report indicating measurable responses to combination chemotherapy with immunotherapy in “recurrent” ovarian SCC. When considered with the findings from previous studies, combination treatment, especially immunotherapy, may lead to improved results. Further studies are needed to confirm our preliminary observations.

## Data Availability Statement

The original contributions presented in the study are included in the article/supplementary material. Further inquiries can be directed to the corresponding author.

## Ethics Statement

The hospital Peking Union Medical College Hospital (CAMS) ethics committee approved this study. The patient signed an informed consent that her data would be used for 40 scientific purposes.

## Author Contributions

XS: manuscript writing. MY: protocol development, manuscript revision, and final approval of the version to be published. YW, D-yC, and J-xY: manuscript revision. All authors listed have made a substantial, direct, and intellectual contribution to the work and approved it for publication.

## Conflict of Interest

The authors declare that the research was conducted in the absence of any commercial or financial relationships that could be construed as a potential conflict of interest.

## Publisher’s Note

All claims expressed in this article are solely those of the authors and do not necessarily represent those of their affiliated organizations, or those of the publisher, the editors and the reviewers. Any product that may be evaluated in this article, or claim that may be made by its manufacturer, is not guaranteed or endorsed by the publisher.
